# Data envelopment analysis to evaluate the efficiency of tobacco treatment programs in the NCI Moonshot Cancer Center Cessation Initiative

**DOI:** 10.1186/s43058-023-00433-3

**Published:** 2023-05-11

**Authors:** Kathryn Pluta, Sarah D. Hohl, Heather D’Angelo, Jamie S. Ostroff, Donna Shelley, Yasmin Asvat, Li-Shiun Chen, K. Michael Cummings, Neely Dahl, Andrew T. Day, Linda Fleisher, Adam O. Goldstein, Rashelle Hayes, Brian Hitsman, Deborah Hudson Buckles, Andrea C. King, Cho Y. Lam, Katie Lenhoff, Arnold H. Levinson, Mara Minion, Cary Presant, Judith J. Prochaska, Kimberly Shoenbill, Vani Simmons, Kathryn Taylor, Hilary Tindle, Elisa Tong, Justin S. White, Kara P. Wiseman, Graham W. Warren, Timothy B. Baker, Betsy Rolland, Michael C. Fiore, Ramzi G. Salloum

**Affiliations:** 1 Department of Health Outcomes and Biomedical Informatics, University of Florida College of Medicine, 2004 Mowry Rd, Gainesville, FL 32610 USA; 2 University of Wisconsin Carbone Cancer Center, 600 Highland Ave, Madison, WI 53705 USA; 3 School of Medicine and Public Health, University of Wisconsin, 750 Highland Ave, Madison, WI 53705 USA; 4 National Cancer Institute, 9609 Medical Center Dr, Rockville, MD 20850 USA; 5 Memorial Sloan Kettering Cancer Center, 1275 York Ave, New York, NY 10065 USA; 6 New York University School of Global Public Health, 708 Broadway, New York, NY 10003 USA; 7 Rush University Medical Center and Rush Cancer Center, 1725 W Harrison St, Suite 1010, Chicago, IL 60612 USA; 8 Washington University Siteman Cancer Center, 4921 Parkview Pl, St. Louis, MO 63110 USA; 9 Medical University of South Carolina, 171 Ashley Ave, Charleston, SC 29425 USA; 10 University of Virginia Comprehensive Cancer Center, 1240 Lee St, Charlottesville, VA 22903 USA; 11 University of Texas Southwestern Medical Center, 5323 Harry Hines Blvd, Dallas, TX 75390 USA; 12 Fox Chase Cancer Center, 333 Cottman Ave, Philadelphia, PA 19111 USA; 13 University of North Carolina Lineberger Cancer Center, 450 West Dr, Chapel Hill, NC 27599 USA; 14 Virginia Commonwealth University Department of Psychiatry, 501 N 2Nd St, Suite 400B, Richmond, VA 23219 USA; 15 Northwestern University Feinberg School of Medicine and Lurie Comprehensive Cancer Center of Northwestern University, 420 E Superior St, Chicago, IL 60611 USA; 16 Indiana University Simon Comprehensive Cancer Center, 535 Barnhill Dr, Indianapolis, IN USA; 17 University of Chicago Medicine Comprehensive Cancer Center, 5758 S Maryland Dr, Chicago, IL 60637 USA; 18 Huntsman Cancer Institute, University of Utah, 1950 Circle of Hope Dr, Salt Lake City, UT 84112 USA; 19 One Medical Center Drive, Dartmouth-Hitchcock Norris Cotton Cancer Center, Lebanon, NH 03756 USA; 20 University of Colorado Comprehensive Cancer Center, 1665 North Aurora Court, Aurora, 200480045 USA; 21 City of Hope Comprehensive Cancer Center and Beckman Research Institute, 1500 E Duarte Rd, Duarte, CA 91010 USA; 22 Stanford Cancer Institute, Stanford University, 265 Campus Dr, Ste G2103, Stanford, CA 94305 USA; 23 H. Lee Moffitt Cancer Center, 3011 Holly Dr, Tampa, FL 33612 USA; 24 Georgetown University Lombardi Comprehensive Cancer Center, 3800 Reservoir Rd NW, Washington, DC, 20007 USA; 25 Vanderbilt University Medical Center Vanderbilt-Ingram Cancer Center, 2220 Pierce Ave, Nashville, TN 37232 USA; 26 University of California Davis Comprehensive Cancer Center, 2279 45Th St, Sacramento, CA 95817 USA; 27 Philip R. Lee Institute for Health Policy Studies, University of California San Francisco, 490 Illinois St, Floor 7, San Francisco, CA 94158 USA; 28 University of Wisconsin Institute for Clinical and Translational Research, 750 Highland Ave, Madison, WI 53705 USA

**Keywords:** Data envelopment analysis, Efficiency, Program performance, Implementation costs, Smoking cessation, Implementation science, Tobacco treatment, Cancer

## Abstract

**Background:**

The Cancer Center Cessation Initiative (C3I) is a National Cancer Institute (NCI) Cancer Moonshot Program that supports NCI-designated cancer centers developing tobacco treatment programs for oncology patients who smoke. C3I-funded centers implement evidence-based programs that offer various smoking cessation treatment components (e.g., counseling, Quitline referrals, access to medications). While evaluation of implementation outcomes in C3I is guided by evaluation of reach and effectiveness (via RE-AIM), little is known about technical efficiency—i.e., how inputs (e.g., program costs, staff time) influence implementation outcomes (e.g., reach, effectiveness). This study demonstrates the application of data envelopment analysis (DEA) as an implementation science tool to evaluate technical efficiency of C3I programs and advance prioritization of implementation resources.

**Methods:**

DEA is a linear programming technique widely used in economics and engineering for assessing relative performance of production units. Using data from 16 C3I-funded centers reported in 2020, we applied input-oriented DEA to model technical efficiency (i.e., proportion of observed outcomes to benchmarked outcomes for given input levels). The primary models used the constant returns-to-scale specification and featured cost-per-participant, total full-time equivalent (FTE) effort, and tobacco treatment specialist effort as model inputs and reach and effectiveness (quit rates) as outcomes.

**Results:**

In the DEA model featuring cost-per-participant (input) and reach/effectiveness (outcomes), average constant returns-to-scale technical efficiency was 25.66 (*SD* = 24.56). When stratified by program characteristics, technical efficiency was higher among programs in cohort 1 (*M* = 29.15, *SD* = 28.65, *n* = 11) vs. cohort 2 (*M* = 17.99, *SD* = 10.16, *n* = 5), with point-of-care (*M* = 33.90, *SD* = 28.63, *n* = 9) vs. no point-of-care services (*M* = 15.59, *SD* = 14.31, *n* = 7), larger (*M* = 33.63, *SD* = 30.38, *n* = 8) vs. smaller center size (*M* = 17.70, *SD* = 15.00, *n* = 8), and higher (*M* = 29.65, *SD* = 30.99, *n* = 8) vs. lower smoking prevalence (*M* = 21.67, *SD* = 17.21, *n* = 8).

**Conclusion:**

Most C3I programs assessed were technically inefficient relative to the most efficient center benchmark and may be improved by optimizing the use of inputs (e.g., cost-per-participant) relative to program outcomes (e.g., reach, effectiveness). This study demonstrates the appropriateness and feasibility of using DEA to evaluate the relative performance of evidence-based programs.

**Supplementary Information:**

The online version contains supplementary material available at 10.1186/s43058-023-00433-3.

Contributions to the literature

- This study demonstrates the utility of data envelopment analysis (DEA) as a novel implementation science tool for evaluating program efficiency.
- DEA allows for the identification of program factors associated with higher levels of relative efficiency, which can be leveraged to increase efficiency across peer programs
- Decision makers can use findings from DEA to improve efficiency of existing tobacco treatment programs within oncology settings by identifying the types of programs that maximize reach and effectiveness while minimizing costs.
- Maximizing efficiency of tobacco treatment programs can promote better program sustainability long term.

## Background

Tobacco use is a preventable risk factor that can exacerbate adverse health outcomes for individuals with cancer, increasing risk for all-cause mortality, cancer-specific mortality, cancer recurrence, and worsening response to cancer treatment [1, 2]. Failed first-line cancer therapy associated with continued tobacco use adds a major burden to the US healthcare system, amounting to approximately $3.4 billion per year, or $10,678 annual cost per patient [3]. Thus, timely tobacco treatment in patients with cancer is imperative to mitigate the harmful effects of tobacco use on individuals’ health and rising health care costs.

The importance of smoking cessation for individuals with cancer is widely recognized by cancer organizations and the Surgeon General [1, 2]. However, patients with cancer face barriers to accessing tobacco treatment as part of their cancer care [1, 4, 5]. Screening patients for smoking using electronic health records and referring them to smoking cessation programs can effectively facilitate their engagement in these programs [6]. However, tobacco cessation interventions are not routinely offered as a part of standard care in oncology. As such, patients with cancer who smoke are not consistently connected with necessary tobacco treatment resources [7]. 

In response to this gap, the Cancer Center Cessation Initiative (C3I) was launched in 2017 as part of the National Cancer Institute (NCI) Cancer Moonshot program with the aim of integrating tobacco cessation treatments into routine cancer care. The goal of this multilevel implementation initiative has been to foster and sustain evidence-based tobacco cessation programs for patients with cancer and to encourage system-level change by identifying and referring patients who use tobacco to cessation treatments [8, 9]. C3I includes 52 NCI-designated cancer centers, which have implemented evidence-based tobacco treatments into their standard of care [10]. C3I provided funding to cancer centers across three cohorts: 42 centers received funding for two years over two cycles (i.e., cohort 1: 2017–2019; cohort 2: 2018–2020), and 10 centers received funding for 1 year (i.e., cohort 3: 2020–2021). Eleven cohort 1 and 2 centers were funded for an additional year as enhancement sites. Each funded cancer center offers evidence-based smoking cessation treatments (e.g., in-person/telephone/video-based/point-of-care counseling, cessation mediation, patient education material, Quitline referral), with variability in the provision of type and number of specific treatments across centers.

C3I is guided by the reach, effectiveness, adoption, implementation, and maintenance (RE-AIM) framework, which facilitates multilevel assessment of the programs’ health-related impact (i.e., individual, organizational, community) [11–13]. Identifying factors that contribute to the fidelity and effectiveness of C3I programs is imperative for their sustainability [14, 15]. Cancer centers participating in C3I reported key implementation outcomes biannually for continued program evaluation and to inform future resource allocation needs.

Program reach and effectiveness are two key outcomes of interest that are reported by C3I-funded cancer centers as standardized outcomes and have been used to evaluate the success of the tobacco treatment programs. Reach is defined as the proportion of patients who received at least one component of evidence-based, tobacco treatment. Effectiveness is defined as patient-reported 7-day point prevalence abstinence at 6-month follow-up. C3I centers’ reach and effectiveness rates varied as a function of center characteristics, including cancer center size, implementation strategies used, and types of treatments offered. For instance, Hohl et al. [16] found that cancer center size (i.e., number of patients served) was positively associated with effectiveness and negatively associated with reach. C3I centers that implemented tobacco treatment programs center-wide had similar effectiveness and higher reach than centers that engaged in partial implementation [16]. Further, centers that offered tobacco treatment through interactive voice response (i.e., automated calls) had the highest median reach and lowest effectiveness, whereas centers that implemented in-person face-to-face counseling had the highest effectiveness but the lowest reach. Additionally, offering six or fewer (vs. seven) types of tobacco treatments within a program was associated with higher reach and effectiveness.

In addition to reach and effectiveness, cost is an important factor that can affect program sustainability [17]. An economic evaluation of 15 C3I sites found that monthly operating costs per site ranged from $5129 to $20,751 (*median* = $11,045), with most costs going towards personnel [18]. Cost per participant ranged from $70 to $2093 (*median* = $454) and cost per quit was less than $3500 across centers. Overall, C3I centers achieved satisfactory quit rates at reasonable costs [14], and the programs were expected to become more cost-effective as they continued to scale up. Identifying factors associated with high reach and effectiveness while minimizing costs is imperative for maximizing program efficiency and sustainability. However, operating costs of C3I programs in relation to their reach and effectiveness has not been examined. Therefore, the objective of this study was to compare program outcomes relative to expended resources and to identify best practices across cancer centers, including which program components and implementation strategies were associated with optimal efficiency.

To compare C3I program outcomes relative to expended resources, we applied a mathematical optimization method called data envelopment analysis (DEA). DEA is widely used in economics and engineering for measuring the relative performance of production units [19, 20]. One advantage of DEA is that it does not require any parametric assumptions regarding data distributions, and data are not restricted to any functional form [20]. DEA assesses the ratio of outputs to inputs when evaluating performance and produces a “best practice frontier” representing the best performing units. Performance of the remaining units is calculated as a relative score compared to the unit(s) located on the best practice frontier. Thus, DEA can be particularly useful in assessing which C3I programs are operating most efficiently. We assessed efficiency of C3I programs to demonstrate the application of this method in implementation science. In this study, each C3I program was compared against the best practice frontier which consists of the C3I program(s) with the most efficient performance (i.e., proportion of observed outcomes to benchmarked outcomes for given input levels). Multiple inputs and outcomes can be considered simultaneously while using DEA, which allows for evaluation of several factors related to performance, such as cost, reach, and effectiveness. Although these implementation outcomes are commonly assessed in implementation research, efficiency is rarely evaluated as it relates to the implementation of evidence-based practices. Thus, this study also seeks to demonstrate the utility of using DEA as a program evaluation tool within the field of implementation science. By assessing the reach and effectiveness relative to resources expended, decision makers can be better informed regarding which factors contribute to the most effective and sustainable program components in order to maximize impact of their programs.

## Methods

### Overview

This is a descriptive study using DEA to examine reach and effectiveness of the C3I program relative to resources expended. DEA applications in health services give insights into which organizations are more efficient than others using program outcomes as outputs and resources expended as inputs [21]. This study examined program efficiency in cohorts 1 and 2 of C3I participating cancer centers that had implemented tobacco treatment into oncology care. C3I sites implemented variations in tobacco treatment components and implementation strategies, requiring investments in different types and proportions of resources, including expenditures on staffing, medications, and electronic health record systems. The heterogeneity of components and implementation strategies, as well as the presence of multiple outcomes of interest, pose challenges for evaluating the relative performance of these programs. Cancer centers with existing tobacco treatment programs have historically focused on different outcomes as their primary objectives (e.g., by emphasizing reach vs. effectiveness) [22]. The diversity in the way centers invest in resources and prioritize outcomes is reflective of the differences in implicit valuation that cancer centers assign to various program components and outcomes. DEA allows for multiple inputs and outcomes to be considered simultaneously without any parametric assumptions on data distributions. DEA is appropriate for comparing C3I programs due to its characterization of the implicit valuation placed on program components, which varies by site, and its ability to simultaneously model efficiency for multiple outcomes, such as reach and effectiveness. Sixteen of 42 NCI-designated cancer centers from cohorts 1 and 2 that had complete data for input and outcome measures of interest (i.e., tobacco treatment specialist (TTS), full time equivalent (FTE) of overall staff, cost-per-patient, reach, and effectiveness) were included in this study. We stratified the analysis by cancer center characteristics because identifying factors associated with cancer centers that maintain high reach and effectiveness given budget constraints is important to foster sustainability of C3I programs. Stratifying the analysis by cancer center characteristics clarifies which components are associated with greater efficiency and informs how efficiency can be improved at underperforming centers.

### Data collection procedures

Tobacco treatment program evaluation data were reported to the C3I Coordinating Center, based at the University of Wisconsin-Madison Carbone Cancer Center. The Coordinating Center assisted grantees with integrating evidence-based tobacco treatment services into cancer care [10] and created standardized metrics to evaluate the tobacco treatment programs. All C3I cancer centers received an online questionnaire via Qualtrics (Provo, UT) every 6 months from the Coordinating Center, which assessed center characteristics (e.g., size, TTS FTE, treatments offered) and outcomes (e.g., reach, effectiveness). Specific methods regarding C3I measurement are detailed elsewhere [10, 23]. C3I centers were given the option to report implementation costs and other resources expended (e.g., number of tobacco treatment specialists, program staff FTE) using an additional biannual Qualtrics survey [14]. Cost data used in this study were reported during the January to June 2020 reporting cycle. To be included in this study, centers must have reported reach, effectiveness, cost, total program FTE, and tobacco treatment specialist FTE. This study was classified by the University of Wisconsin-Madison and University of Florida Institutional Review Boards as program evaluation and therefore exempt.

### Site characteristics

Data reported included: size of the cancer center, smoking prevalence for patients at the center, presence of a point-of-care tobacco cessation intervention (i.e., in-person, telehealth) [24], and whether sites were part of the first or second C3I cohort. Cancer center size was assessed by number of unique adult cancer patients served by the center during the 6-month reporting period. Smoking prevalence was assessed by the proportion of cancer patients within the center who were documented in the electronic health record system as currently smoking cigarettes. Centers that offered point-of-care counseling for tobacco cessation (i.e., in-person or telehealth) included programs with a brief intervention delivered by a health care provider during routine oncology appointments to discuss evidence-based tobacco treatment options and offer tobacco cessation-related advice [10, 16, 23]. C3I programs were also categorized by cohort, whereby cohort 1 sites received funding from 2017 to 2019, and cohort 2 sites received funding between 2018 and 2020. The reporting period was the same for both cohorts, and we did not control for lead time among cohort 1 sites. Therefore, cohort 1 sites had more implementation experience than cohort 2 sites, on average, for each assessment.

### Tobacco treatment program components

C3I sites reported the types of evidence-based treatments offered in their programs. These treatments included the following: in-person individual or group counseling, telephone-based counseling, point-of-care counseling, interactive voice response system track and triage services (i.e., TelASK), Quitline referral, SmokefreeTXT text messaging service, online resources (e.g., smokefree.gov), and smoking cessation medications.

### Input measures

Measures indicating presence of a TTS on site, total FTE, and cost were collected through the biannual cost surveys and used as input measures. C3I cancer centers reported FTE of tobacco treatment specialists employed in the program. Sites also reported FTE associated with all tobacco treatment program staff by personnel type, which was summed across personnel types to derive the total FTE measure. “Other personnel FTE” was calculated by subtracting TTS FTE from total FTE. We calculated cost-per-patient by dividing total monthly operating costs of each participating C3I center by the number of patients participating in a tobacco treatment program within the 6-month reporting period. Details of how total monthly operating costs were calculated can be found elsewhere [14]. 

### Outcome measures

Reach was assessed as the proportion of unique patients seen during the 6-month reporting period who used tobacco and received at least one type of evidence-based tobacco treatment (e.g., tobacco cessation medications, Quitline referral, point-of-care counseling [24]), relative to the total number of patients who smoked at each C3I center. Effectiveness was assessed as the proportion of patients currently using tobacco who engaged in tobacco treatment and reported abstinence from tobacco use for a minimum of seven days at six months follow-up. The number of total patients using tobacco was assessed using two items on the C3I 6-month survey: (1) for how many patients who received tobacco treatment in the July–December 2020 reporting period do you have follow-up effectiveness data? (2) For how many patients who received tobacco treatment in the July-December 2020 reporting period are 6-month effectiveness data missing? While a small number of programs implemented biochemical verification as part of their assessments, this was not standard across all programs. Therefore, we only used self-reported abstinence for our assessments of program effectiveness. A complete response approach was used wherein each center determined their own denominator for effectiveness based on their center’s reporting practices.

### Assessment of program performance

We applied DEA to assess the relative performance of C3I centers [20]. We used the DEA optimization method, which has been applied to estimate the technical or cost efficiency of healthcare programs [25–27]. DEA determines how efficiently a program converts inputs into outcomes compared with other programs and produces a best practice frontier comprising the most efficient programs.

### Efficiency scores

We used DEA to estimate efficiency scores for each program as the ratio of the weighted sum of outcomes to the weighted sum of inputs, and graphically plotted the efficiency scores according to cost and reach/effectiveness. We applied the input-oriented DEA approach with constant returns-to-scale [19, 28]. Under the input orientation, the efficiency measure is based on the proportion to which the observed input levels can be produced for given outcome levels. Compared to efficiency scores, rankings are robust as they are not based on unstable solutions of linear programming models. We compared efficiency scores across subgroups of sites, by funding cycle, core components, and implementation strategies used. The most efficient program(s) are used as the benchmark for comparison with other programs. The efficiency of any program is relative to the efficiency of other programs in the sample, and the relative efficiency of any given program can change when compared to a different set of programs.

### Slacks

Slacks represent excess input utilization or shortages in outcomes within DEA [29]. We assessed the mean amount of slack among inefficient C3I programs, relative to the most efficient program(s), for each input and outcome (i.e., distance between inefficient programs and the most efficient program). We reported the percentage of change needed to eliminate inefficiencies and to achieve performance consistent with the most efficient program(s) on the best practice frontier.

### Analyses

Three DEA models assessed the relative efficiency of the sixteen C3I programs with complete data.

- Model 1 input: cost per participant; outcomes: reach, effectiveness.
- Model 2 inputs: TTS, other personnel; outcomes: reach.
- Model 3 inputs: TTS, other personnel; outcomes: effectiveness.

Analyses were also stratified by C3I center characteristics. We conducted all analyses using the PIM-DEA V.3.2 software.

## Results

Table 1 includes descriptive statistics summarizing site characteristics, inputs, and outcomes of the included C3I centers. Cancer centers served an average of 24,652 (*standard deviation, SD* = 21,596, *median* = *22,075, range* = 507–89,485) patients during the 6-month reporting period, and median smoking prevalence was 9.3% (*range* = 2.2–47.1%) across centers; 44% of cancer centers had implemented point-of-care interventions and 69% were part of cohort 1 (vs. cohort 2). Mean number of tobacco treatment specialist FTE was 0.66 (*SD* = 0.60), and mean total FTE was 1.39 (*SD* = 0.74). Average cost-per-patient was $572 (*SD* = $518, *median* = $474), and average cost-per-quit was $2981 (*SD* = $2015, *median* = 2765). Overall, programs reached 24.4% of patients who smoked (*SD* = 14.1, *range* = 2.5–47.8%) and had a 20.4% effectiveness (*SD* = 10.6, *range* = 2.6–35.3%), on average.

**Table 1:** Site characteristics, inputs and outputs of the C3I programs (*n* = 16)

**Site characteristics**	**Median**	**Mean**	**SD**	**Min**	**Max**
Cancer center size (number of patients served)	22,075	24,652	21,596	507	89,485
Smoking prevalence	6.5%	9.3%	10.5%	2.2%	47.1%
	**N (%)**				
Point of care intervention	7 (44%)				
Cohort 1 (vs. cohort 2)	11 (69%)				
**Inputs**	**Median**	**Mean**	**SD**	**Min**	**Max**
Tobacco treatment specialist	0.65	0.66	0.60	0.00	2.00
Total FTE	1.31	1.39	0.74	0.42	2.42
Cost per patient	$454	$572	$518	$70	$2093
Cost per quit	$2765	$2981	$2015	$330	$9628
**Outputs**	**Median**	**Mean**	**SD**	**Min**	**Max**
Reach	108.0	254.13	257.03	46	935
Reach percent	25.0%	24.4%	14.1%	2.5%	47.8%
Effectiveness	33.0	38.3	44.6	7	197
Effectiveness percent	19.9%	20.4%	10.6%	2.6%	35.3%

*SD* Standard deviation

**Fig. 1:**
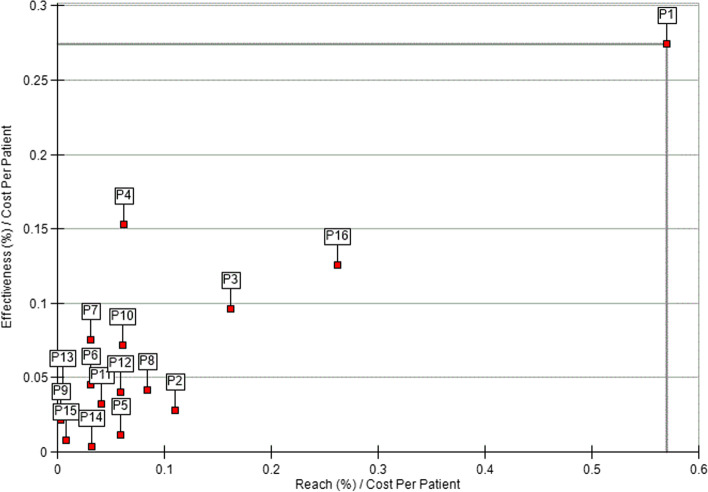
Efficiency frontier for C3I programs: reach (%) and effectiveness (%) relative to cost-per-participant (*n* = 16). P1 is on the best practice frontier (i.e., the most efficient unit)

In the first DEA model (Fig. 1), we assessed reach and effectiveness (as outcomes) relative to cost-per-participant (as the input). Only one program was located on the best practice frontier (i.e., benchmark program(s) with the most efficient performance), while the majority of programs clustered near the origin (i.e., away from the best practice frontier). This distribution suggests generally low effectiveness and reach relative to costs, in comparison to the one program on the best practice frontier. Six programs had relatively higher effectiveness (vs. reach), and 10 programs had relatively higher reach (vs. effectiveness).

Next, we used DEA to assess effectiveness and reach relative to total costs, stratified by program characteristics (Supplementary Materials). C3I centers in cohort 1 were less clustered around the origin than centers in cohort 2, and the best practice frontier was farther from the origin for cohort 1 (vs. cohort 2) (Supplementary Fig. 1). Only one C3I center was located on the best practice frontier for each cohort. Similarly, C3I centers that did not implement point-of-care interventions were clustered closer to the origin, suggesting that point-of-care was associated with higher reach and effectiveness relative to costs (Supplementary Fig. 2). Only one C3I center was located on the best practice frontier for each model assessing point-of-care. Larger cancer centers (i.e., above median size) were more efficient and had generally greater reach than smaller centers (Supplementary Fig. 3). One C3I center was located on the best practice frontier in each model assessing larger and smaller centers. Centers with below-median smoking prevalence were clustered more closely to the origin, suggesting lower reach and effectiveness relative to total cost than centers with above-median smoking prevalence (Supplementary Fig. 4). One C3I center was located on the best practice frontier in the model for greater than median smoking prevalence, and two C3I centers were located on the best practice frontier in the model for lower than median smoking prevalence.

**Fig. 2:**
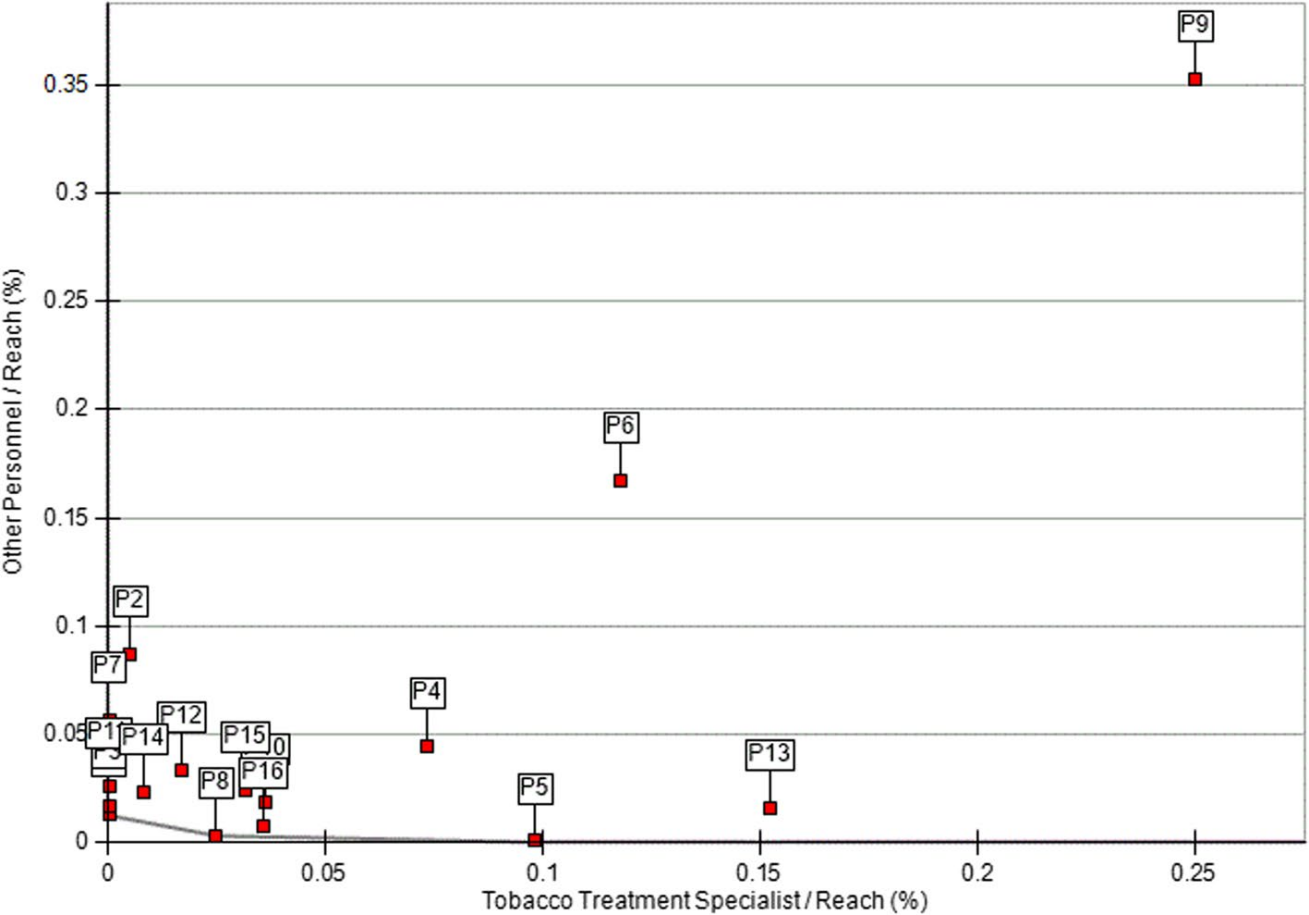
Efficiency frontier for C3I programs: TTS and other personnel relative to reach (*n* = 16). P9 is on the best practice frontier (i.e., the most efficient unit)

**Fig. 3:**
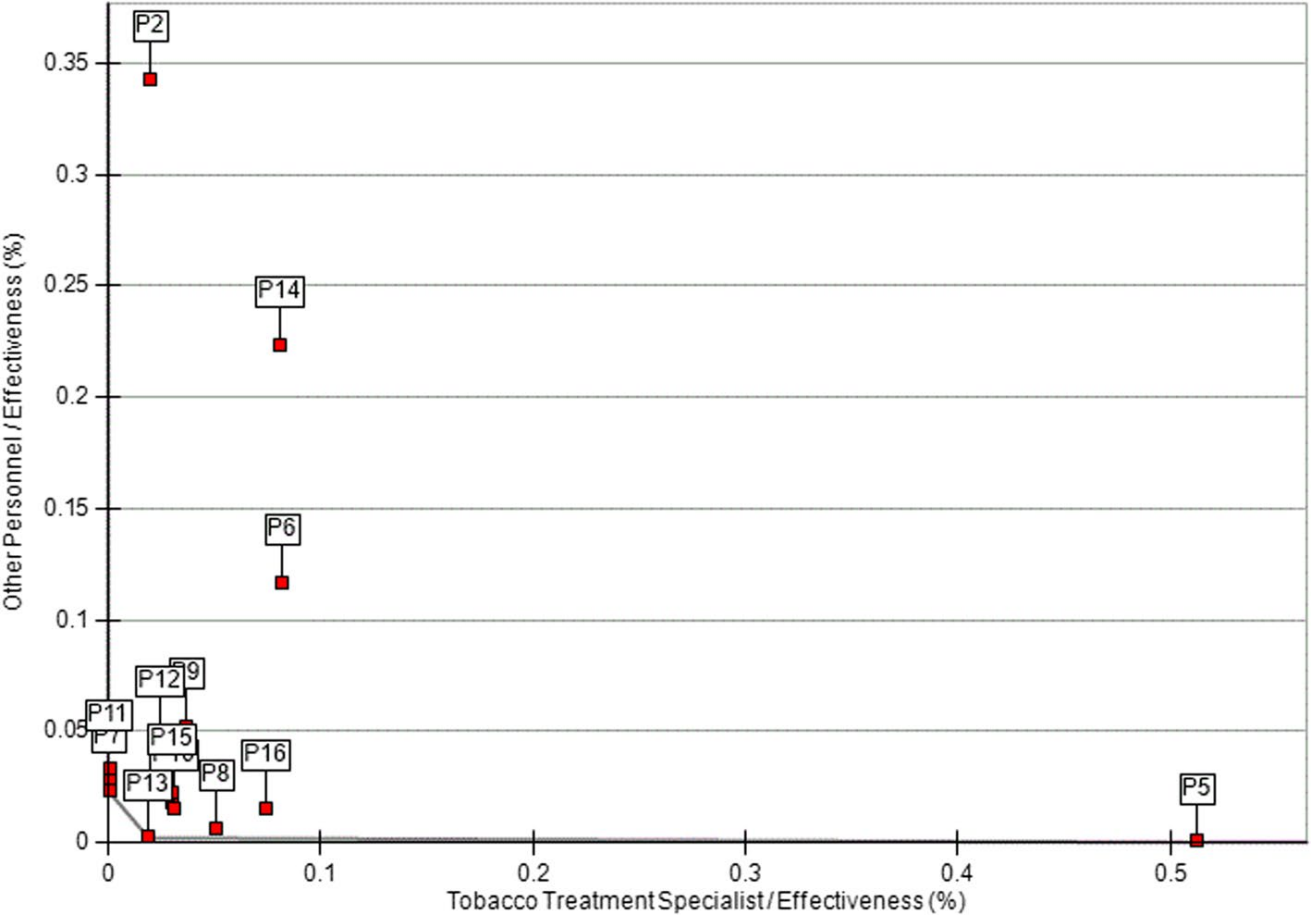
Efficiency frontier for C3I programs: TTS and other personnel relative to effectiveness (*n* = 16). P2 and P5 are on the best practice frontier (i.e., the most efficient units)

Additionally, we used DEA to assess the reach and effectiveness of C3I centers relative to the personnel involved in tobacco treatment administration (i.e., TTS, other personnel). For the first set of models, the inputs were TTS and other personnel, and the output was reach. C3I centers clustered around the origin, suggesting that most had generally low other personnel and TTS relative to reach. Only one C3I center was located on the best practice frontier. Nine C3I centers had higher FTE for other personnel (vs. TTS) relative to reach, whereas seven C3I centers had higher TTS FTE (vs. other personnel) relative to reach (Fig. 2). Next, we used DEA to assess the effectiveness of C3I centers relative to personnel involved in tobacco treatment administration (i.e., other personnel, TTS). C3I centers were clustered near the origin, suggesting lower use of TTS and other personnel relative to effectiveness. Two C3I centers were located on the best practice frontier. Seven C3I centers had higher use of other personnel relative to effectiveness, whereas six centers had higher use of TTS relative to effectiveness (Fig. 3).

Table 2 shows the average efficiency scores overall, by cohort, by whether the program had a point-of-care intervention, by cancer center size, and by smoking prevalence. Sites in cohort 1, those with point-of-care interventions, those within larger-than-median cancer centers, and those with higher-than-median smoking prevalence had higher efficiency scores, on average.

**Table 2:** Technical efficiency scores and returns to the scale of tobacco treatment programs in C3I

	CRS technical efficiency	VRS technical efficiency	Scale efficiency	CRS, *N* (%)	DRS, *N* (%)
All programs (*n* = 16)					
Mean	25.66	76.82	31.40	7 (44)	9 (56)
SD	24.56	22.98	23.73		
Min	2.91	39.2	5.88		
Cohort 1 (*n* = 11)					
Mean	29.15	80.46	32.54	5 (45)	6 (55)
SD	28.65	23.21	26.92		
Min	2.91	46.75	5.88		
Cohort 2 (*n* = 5)					
Mean	17.99	68.82	28.88	2 (40)	3 (60)
SD	10.16	22.76	16.99		
Min	7.94	39.20	10.26		
No point-of-care (*n* = 7)					
Mean	15.59	62.07	24.01	4 (57)	3 (43)
SD	14.31	23.07	15.56		
Min	2.91	39.20	5.88		
Point-of-care (*n* = 9)					
Mean	33.50	88.30	37.15	3 (33)	6 (67)
SD	28.63	15.89	28.10		
Min	7.94	57.79	10.26		
Center size: < 22,075 (*n* = 8)					
Mean	17.70	74.88	22.38	4 (50)	4 (50)
SD	15.00	22.70	16.41		
Min	2.91	46.75	5.88		
Center size: ≥ 22,075 (*n* = 8)					
Mean	33.63	78.76	40.42	3 (38)	5 (62)
SD	30.38	24.66	27.41		
Min	7.94	39.20	10.26		
Smoking prevalence: < 6.5% (*n* = 8)					
Mean	21.67	72.83	29.03	3 (38)	5 (62)
SD	17.21	23.00	18.69		
Min	2.91	39.20	5.88		
Smoking prevalence: ≥ 6.5% (*n* = 8)				4 (50)	4 (50)
Mean	29.65	80.81	33.77		
SD	30.99	23.80	29.06		
Min	5.69	46.75	10.69		

*CRS* Constant returns to scale, *DRS* Decreasing returns to scale, *SD* Standard deviation, *VRS* Variable returns to scale

The performance analysis identified the slacks, representing either excess input utilization or shortages of output production. Table 3 shows the average slack in programs deemed inefficient. These results represent the combined scores of slack for all inefficient programs, for each input and output. Table 3 also shows the percentage of change in the number of inputs or outputs needed to eliminate the inefficiencies and achieve target levels. Based on our preliminary sample, cost per participant should be reduced by an average of 74.34%, TTS FTE should be reduced by an average 10.98%, and other personnel FTE by 52.18% to maximize efficiency.

**Table 3:** Slacks evaluation for inefficient programs (*n* = 16)

	**Mean (SD)**	**Percentage of change**
**Input slacks**		
Tobacco treatment specialist	0.11 (0.30)	– 10.98
Other personnel	0.15 (0.39)	– 52.18
Cost per participant	597.08 (660.76)	– 74.34
**Output slacks**		
Reach (percent)	30.14 (20.07)	267.67
Effectiveness (percent)	5.31 (3.90)	38.22

*SD* standard deviation

## Discussion

This study demonstrated utility of DEA for implementation research by assessing reach and effectiveness of tobacco treatment programs within NCI-designated cancer centers relative to their operating costs. We identified factors associated with the most optimal programs that could be leveraged to increase efficiency of tobacco treatment programs across centers that function less optimally. Programs that were in cohort 1 (i.e., typically more advanced in implementation), programs that had implemented point-of-care interventions, and programs in cancer centers that were larger in size tended to be more efficient.

This information is particularly useful for program evaluation because it directly compares how well C3I programs converted their available resources into measurable outcomes (i.e., reach, effectiveness). Variability in efficiency was high across C3I centers, which is unsurprising given that some centers had existing infrastructure for tobacco treatment programs, whereas others implemented these programs for the first time. Existing program infrastructure may have contributed to program efficiency, given that these sites would have already implemented some tobacco treatment program-related protocols into their workflows. There was also variability in how long tobacco treatment programs had been implemented across centers. Thus, DEA is an important tool for program evaluation because it can identify which programs effectively maximize their resources given budget constraints. Identifying how resources can be allocated to foster sustainability of C3I centers has implications for other tobacco cessation programs in oncology settings. For instance, DEA may be used to characterize treatment efficiencies in others areas of oncology practice, such as value-based care, enrollment in clinical trials, and improving palliative care and cancer survivorship.

Although research using DEA to assess tobacco treatment programs for oncology patients is limited, DEA has been applied in other healthcare settings. For example, DEA has been applied in examining the efficiency of primary healthcare centers, including inputs and outputs such as number of patients and staff, costs, procedures, prescriptions, and referrals [30]. Additionally, DEA has been used to assess public health concerns regarding healthcare systems and the optimal organization of primary care service delivery, using inputs such as primary care governance, workforce development, and economic conditions, and outputs such as comprehensiveness, access, coordination and service delivery indicators of access continuity and comprehensiveness of care [30]. Moreover, application of DEA is not limited to assessing efficiency of programs and systems, and it has been used to support decision-making in clinical settings. For example, DEA was used for real-time benchmarking in radiotherapy treatment planning, where it was associated with improvement of most treatment plans [31]. Thus, DEA has a broad range of applications within healthcare, including within the oncology domain.

DEA can be used as a stand-alone analysis, given its unique ability to assess the relative efficiency of production units. DEA has been widely applied in other fields such as economics [19, 20] as well as in clinical settings as described above. DEA can also be used in combination with other methods, (e.g., qualitative interviews, longitudinal surveys), to glean a more holistic perspective regarding how to improve program efficiency. For example, conducting qualitative interviews or surveys with personnel directly involved with the implementation procedures could elucidate specific recommendations regarding how to improve efficiency, beyond which inputs and outputs are affecting efficiency [16]. As such, DEA can be used either independently to assess program efficiency or complimentarily with other analyses.

Overall, we found that many C3I sites had low efficiency relative to the best practice frontier. In the DEA model assessing reach and effectiveness relative to program costs, only one program was located on the best practice frontier, and this program appeared to achieve substantially higher efficiency compared to other programs. We examined additional DEA models stratified by key organizational characteristics. C3I centers that were part of cohort 1 (vs. cohort 2), had deployed point-of-care tobacco cessation interventions (vs. no point-of-care), were larger (vs. smaller) in size, and had higher (vs. lower) smoking prevalence tended to be more efficient (i.e., greater reach and effectiveness relative to cost). Thus, centers may prioritize implementing point-of-care interventions over other types of tobacco treatment interventions to maximize efficiency. Although point-of-care interventions may be expensive, it is noteworthy that their implementation was associated with greater program efficiency (i.e., ratio of reach and effectiveness relative to costs). Similarly, larger NCI-designated cancer centers generally achieved higher reach and effectiveness while mitigating costs, suggesting that C3I programs are more sustainable when implemented in larger (vs. smaller) cancer centers, and that smaller cancer centers or community oncology practices may require more resources to sustainably implement tobacco treatment programs.

Additionally, centers that had higher smoking prevalence were generally higher in efficiency. It is possible that centers with lower smoking prevalence were less efficient because there were fewer eligible patients to enroll in the program. C3I centers with lower smoking prevalence had lower reach relative to effectiveness, which suggests that these programs may be underutilized and consequently operate less efficiently than programs with higher enrollment rates. This information can be particularly useful for establishing tobacco cessation programs for individuals with cancer. Selecting locations that are more likely to maintain low costs relative to reach and effectiveness may increase the likelihood that cancer centers will sustain these programs long-term. However, this practice may come at the cost of marginalizing patients in settings with limited resources, which may require greater costs to implement and sustain tobacco treatment programs.

Moreover, whether C3I centers had existing infrastructure for tobacco cessation treatments prior to the initiative may have impacted relative program efficiency. For instance, centers that had independently focused on promoting tobacco cessation programs before joining C3I may be more efficient than centers that initiated tobacco cessation programs as part of C3I. Implementation readiness has been shown to be associated with higher chances that a cancer center provides tobacco cessation treatments to its patients [32]. Thus, the stage of program implementation is another important factor that may contribute to a center’s efficiency and sustainability.

Further, programs that employed less TTS FTE and other personnel FTE on average achieved greater efficiency. Previous research regarding the effects of a TTS for tobacco cessation are mixed. Recent studies found that C3I centers with lower TTS-to-patient ratios tended to have higher reach and lower effectiveness [16] and that counseling delivered by TTSs was associated with higher smoking cessation rates [33]. Future research should investigate whether reducing TTS and personnel is associated with greater efficiency among C3I centers in general, or whether centers with particular characteristics may benefit from an increase, in TTS and other personnel (e.g., large centers and/or those with particularly high smoking prevalence and less program staff). More research is needed to identify the most efficient TTS staff-to-patient ratio and explore the contexts in which TTS and other personnel are essential for maximizing program efficiency.

It is important to note that it can be challenging to find a balance between maximizing research and delivering an effective intervention, particularly in the oncology setting. Individuals who continue smoking after their cancer diagnosis can be especially difficult to treat even with a high intensity intervention. Therefore, assessing the external validity and cumulative impact of smoking cessation interventions in oncology settings is of utmost importance. We assessed reach and effectiveness as separate outcomes, however, both must be considered to assess population impact. The cumulative impact of an intervention is a function of every step of dissemination and participation (e.g., proportion of staff that take part, patients that accept participation, patients that benefit from the intervention and continue benefitting 6 months later) [34]. Even interventions that have high effectiveness may yield low population impact after accounting for participation and retention issues at every level of dissemination. As such, consistent and transparent reporting about participation and representativeness at all levels of dissemination are vital for evaluating the cumulative impact of interventions. Future research should evaluate the cumulative impact of C3I programs on tobacco cessation outcomes across various contexts.

## Limitations

This study is not without limitations. First, our convenience sample of 16 NCI-designated C3I centers may not be representative of many cancer care programs. This sample consisted of cancer centers that received supplemental funding to improve or expand tobacco cessation resources; thus, results may not be generalizable to other cancer centers and should be interpreted with caution. The programs in this sample reported low proportions of individuals who were American Indian or Alaska Native (≤ 1%), Asian, Native, or Pacific Islander (≤ 1%), or Hispanic (3%); therefore, generalization of findings to these populations may be limited. However, smoking prevalence among C3I centers was similar to estimates of national rates of tobacco use among individuals who have had cancer [35–37]. 

It is possible that the results do not accurately represent the experiences with efficiency across all tobacco treatment programs in C3I. Therefore, a larger sample of C3I centers is needed before factors affecting program efficiency can be reliably assessed and interpreted. Despite this limitation, we achieved the primary goal of this study, which was to demonstrate the benefits of using DEA as a tool for assessing program implementation and performance. Specifically, we showed that DEA can be used to inform program efficiency by assessing readily available practice parameters, such as program reach, effectiveness, and cost.

Another limitation is the reporting of outcomes and program features was voluntary; therefore, data collected from C3I centers may be partially incomplete. Missing data, whether deliberate or coincidental, can skew findings [38]; therefore, more automated data reporting of tobacco treatment program measures would improve future data quality. There was also a lack of uniformity regarding which program personnel reported data to C3I, which may have resulted in between-reporter inconsistencies. Each center also determined their own denominator for effectiveness based on their center’s reporting practices, which exacerbates variability in reporting across centers.

Finally, some of the data for this study were collected during the COVID-19 pandemic, when many non-emergent appointments were postponed or canceled. Other pandemic-related changes, such as limited staff due to illness, staff changes, and program changes with implementation of telehealth, may have impacted program costs and efficiency. Reach and effectiveness may have been affected during this time due to pandemic-related restrictions and barriers. On the other hand, reach may have increased with the pandemic related transformation to telehealth treatment models. Data were reported at the level of the C3I center; therefore, we did not have access to individual-level data. Consequently, we were unable to investigate more granular factors that may be associated with program efficiency, such as which specific tobacco treatments patients were receiving, at what frequency, and whether efficiency was moderated by patient characteristics (e.g., age, cancer site, treatment) [35]. Given these restrictions, we were also unable to undertake a thorough analysis of potential confounding factors that may account for observed relationships, such as differences in the types of patients who received different treatments, and effects of other program features that were not measured or reported.

## Conclusion

DEA is a useful tool for assessing the relative efficiency of organizations that implement evidence-based programs in a way that is not possible with other analytic methods. In the case of C3I, identifying factors associated with high reach and effectiveness, while maintaining low operating costs is important for the sustainability of tobacco treatment programs. Decision makers can use findings from DEA to improve efficiency of existing tobacco treatment programs within oncology settings and evaluate how cancer centers could most effectively support implementation of tobacco treatment programs. This study demonstrated that DEA provides valuable information that can foster more sustainable implementation of tobacco treatment programs in oncology settings.

## Supplementary Information

**Additional file 1:** **Supplementary Figure 1.** Efficiency frontier for C3I programs in Cohort 1 (left) and Cohort 2 (right): reach and effectiveness relative to cost-per-participant. **Supplementary Figure 2.** Efficiency frontier for C3I programs with (left) and without (right) a point-of-care intervention: reach and effectiveness relative to cost-per-participant. **Supplementary Figure 3.** Efficiency frontier for C3I programs at centers larger (left) and smaller (right) than median size: reach and effectiveness relative to cost-per-participant. **Supplementary Figure 4.** Efficiency frontier for C3I programs at centers with higher (left) and lower (right) than median smoking prevalence: reach and effectiveness relative to cost-per-participant.

## Data Availability

The datasets generated and analyzed during the study are not publicly available due to the sensitive nature of some data. The cost dataset is available from the corresponding author upon reasonable request. All other data are available from the C3I coordinating center on reasonable request.

## Abbreviations

C3I: Cancer Center Cessation Initiative
NCI: National Cancer Institute
TTS: Tobacco treatment specialist
FTE: Full time equivalent
