# Supplementary material for: Data envelopment analysis to evaluate the efficiency of tobacco treatment programs in the NCI Moonshot Cancer Center Cessation Initiative
Source: Implement Sci Commun. 2023 May 11;4:50. doi: 10.1186/s43058-023-00433-3 (PMC10173908; doi:10.1186/s43058-023-00433-3)

**Supplementary Materials**

Supplementary Figure 1. Efficiency frontier for C3I programs in Cohort 1 (left) and Cohort 2 (right): reach and effectiveness relative to cost-per-participant.


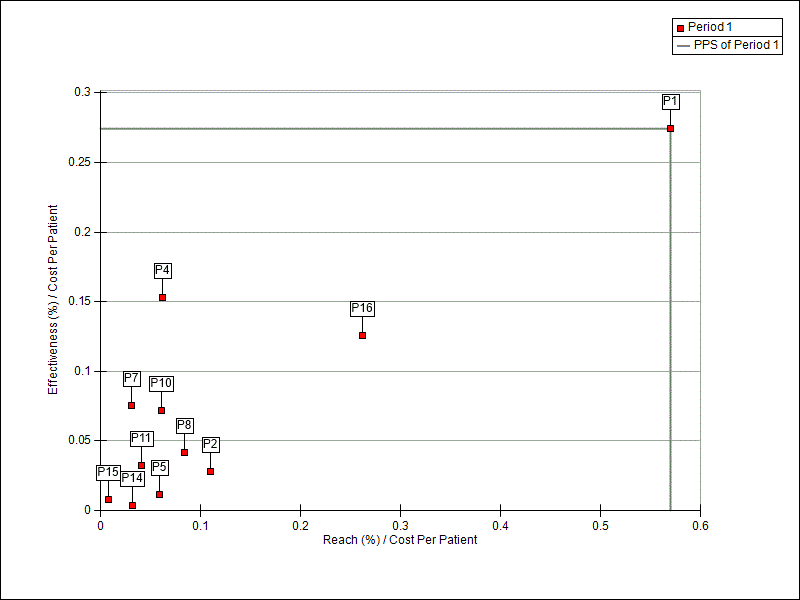

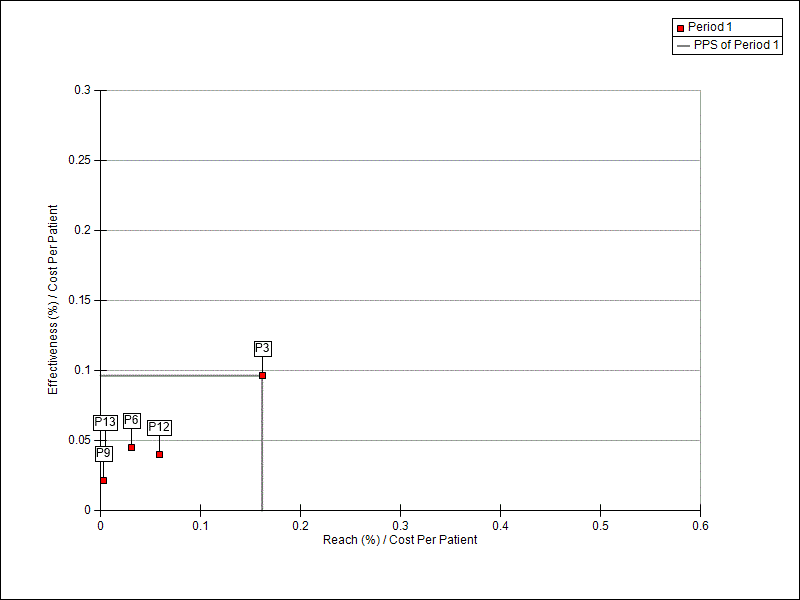


Supplementary Figure 2. Efficiency frontier for C3I programs with (left) and without (right) a point-of-care intervention: reach and effectiveness relative to cost-per-participant.


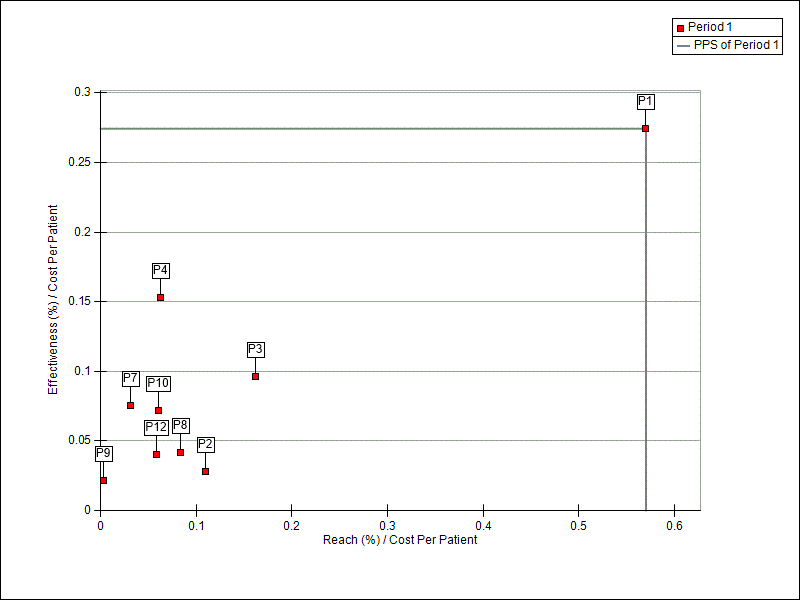

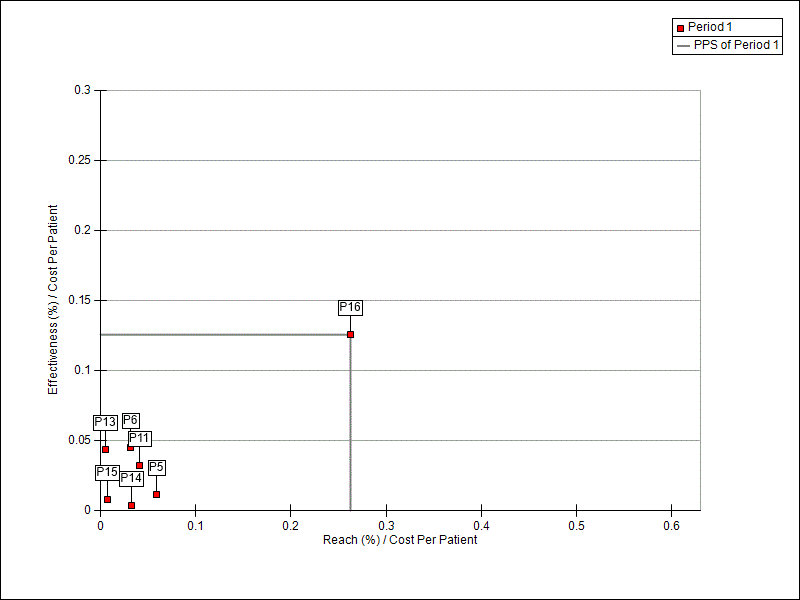


Supplementary Figure 3. Efficiency frontier for C3I programs at centers larger (left) and smaller (right) than median size: reach and effectiveness relative to cost-per-participant.


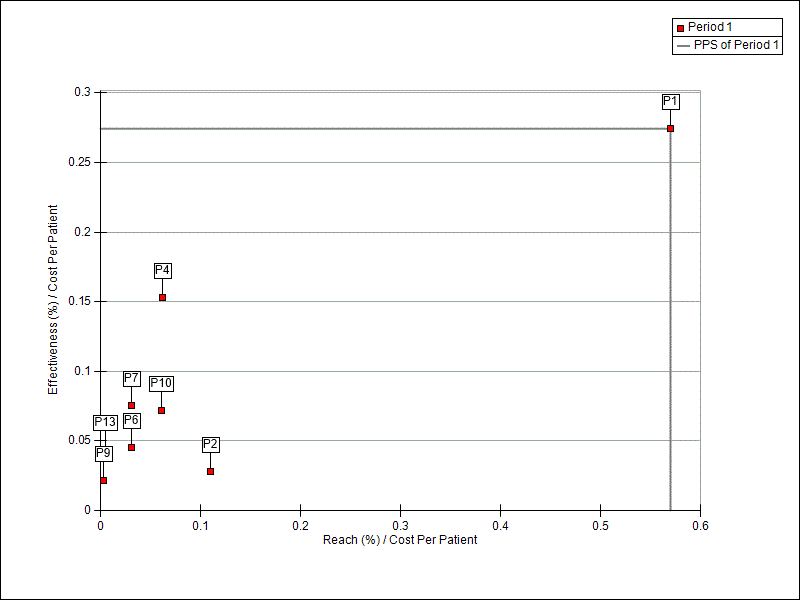

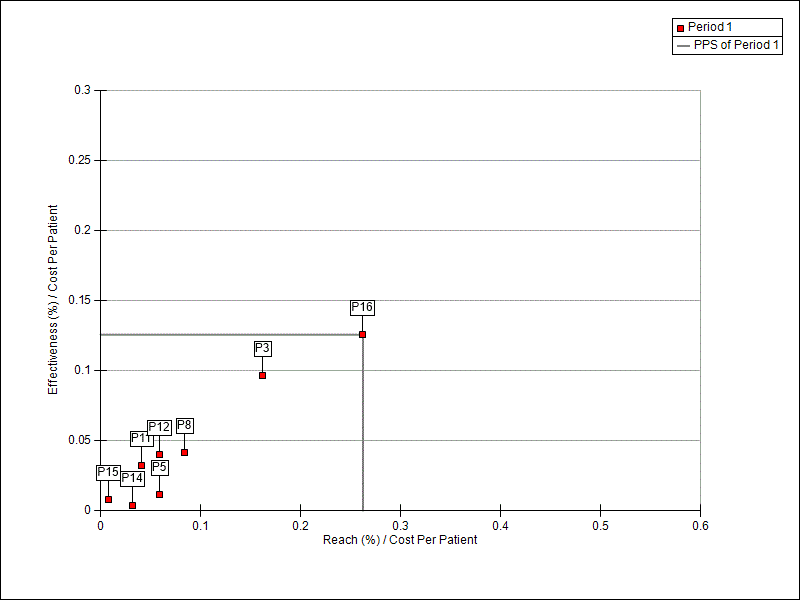


Supplementary Figure 4. Efficiency frontier for C3I programs at centers with higher (left) and lower (right) than median smoking prevalence: reach and effectiveness relative to cost-per-participant.


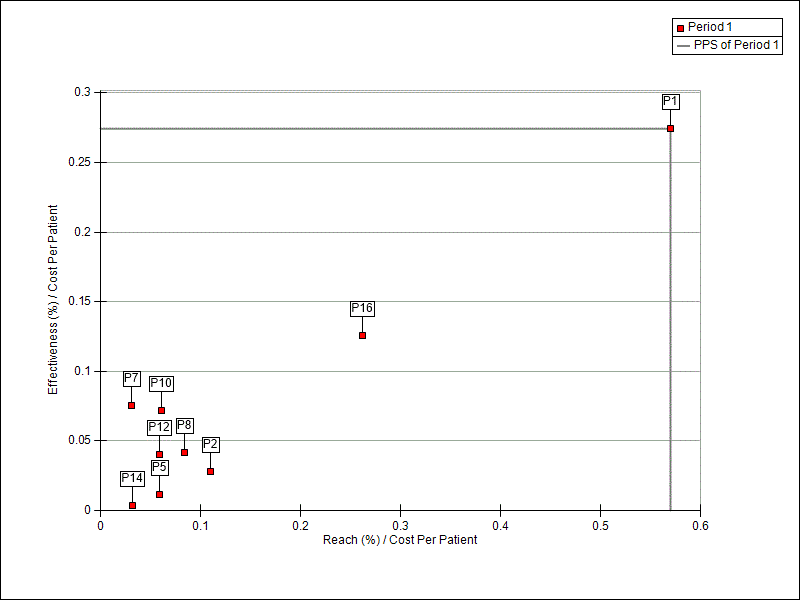

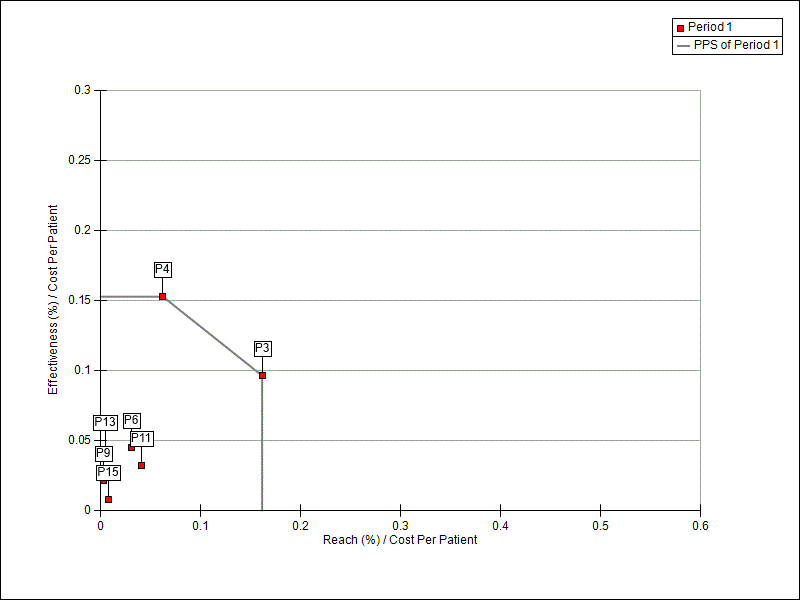

Supplement: Supplementary file 1 — Additional file 1: Supplementary Figure 1. Efficiency frontier for C3I programs in Cohort 1 (left) and Cohort 2 (right): reach and effectiveness relative to cost-per-participant. Supplementary Figure 2. Efficiency frontier for C3I programs with (left) and without (right) a point-of-care intervention: reach and effectiveness relative to cost-per-participant. Supplementary Figure 3. Efficiency frontier for C3I programs at centers larger (left) and smaller (right) than median size: reach and effectiveness relative to cost-per-participant. Supplementary Figure 4. Efficiency frontier for C3I programs at centers with higher (left) and lower (right) than median smoking prevalence: reach and effectiveness relative to cost-per-participant. [file 43058_2023_433_MOESM1_ESM.docx]
